# Fecal transplantation – the new, inexpensive, safe, and rapidly effective approach in the treatment of gastrointestinal tract diseases

**Published:** 2016

**Authors:** R Oprita, M Bratu, B Oprita, B Diaconescu

**Affiliations:** * “Carol Davila” University of Medicine and Pharmacy, Bucharest, Romania

**Keywords:** Clostridium difficile infection, fecal transplant, gastrointestinal tract diseases, FMT

## Abstract

**Introduction.** Fecal transplantation was shown to effectively reduce the reoccurrence in patients with refractory Clostridium difficile infection. New data suggest that fecal transplantation could also be efficient in other gastrointestinal diseases, for instance in inflammatory bowel disease, irritable bowel syndrome, but, there are also some data that could imply the efficacy outside the gastrointestinal tract. Fecal transplantation should be considered a unique agent, capable of treating severe diseases, with essentially no adverse reactions, presenting a cure rate of over 90%.

**Materials and methods.** This prospective study included 33 patients, of whom 28 patients with recurrent or resistant Clostridium difficile infection, who failed to be treated with conventional therapy, which presupposed vancomycin administration and 5 patients with inflammatory bowel disease, more precisely with ulcerative colitis, refractory on biologic agents (infliximab and adalimumab). In most of the cases, fecal transplant was realized with the infusion of stool through colonoscopy.

**Results.** Most of the patients from both groups (Clostridium difficile infection and Ulcerative Colitis) responded (31 patients) with a total relief of the symptoms, after 1 FMT for Clostridium difficile group and after more than one for the ulcerative colitis group. The so-called primary cure rate was 96.42% for Clostridium group. For ulcerative colitis, group 3 of the patients needed 3 or 4 infusions for symptom relief. One patient was categorized as non-responsive (patient with UC) and needed surgery. Due to non-fecal transplant related causes, one death was reported.

**Conclusions.** Fecal transplant is highly effective, safe, with practically no adverse effects, inexpensive, a procedure easy to be done that could be introduced in Clostridium difficile treatment protocols. As for ulcerative colitis treatment with FMT, future randomized controlled trials are needed to prove its efficiency.

## Introduction

Fecal Microbiota transplantationation (FMT) was first described in the Chinese literature when Li Shizhen treated gastrointestinal diseases with “fresh yellow soup” which contained raw fecal [**1**]. Also, during the World War II, German soldiers were treated in Africa with FMT for dysentery [**2**]. In medical literature, Ben Eiseman, a surgeon from Colorado-USA, treated patients with pseudomembranous colitis using fecal enemas [**3**]. Fecal Microbiota transplantationation has gained popularity during the epidemic appearance of Clostridium difficile infection resistant to usual treatments (oral Vancomycin or Metronidazole) and also because of its reduced costs. In 2013, FDA has regulated human feces as an approved drug for treating Clostridium difficile infection. 

Clostridium difficile is the main causative organism of post antibiotic diarrheas. Colonization and becoming a pathogen is facilitated by the disruption of normal intestinal flora due to antimicrobial therapy. It has exotoxins that are responsible for its main symptoms. From 2003 to 2006, C. difficile infections were observed to be more frequent, severe, refractory to standard therapy, and likely to relapse than previously described [**4**]. In 2011, 453.000 cases of Clostridium difficile were diagnosed in the United States and 83,000 episodes were first recurrences, and 29,300 patients died [**5**].

FMT has been experimentally used to treat other gastrointestinal diseases, including colitis, constipation, irritable bowel syndrome, and neurological conditions such as multiple sclerosis and Parkinson disease. Published experience of ulcerative colitis treatment with FMT largely showed that multiple and recurrent infusions are required to achieve prolonged remission or “cure” [**6**]. Studies of the gut microbiome in IBD have identified a variety of changes in the intestinal bacteria in patients including a decreased bacterial diversity, and more bacterial instability than it was seen in healthy individuals. Newer hypotheses about the pathogenesis of IBD now involve a complicated interactive network between host genetic factors and the gut microbiome, which results in loss of the homeostatic mechanisms between mucosa and the microflora [**7**]. 

**Aim**

The aim of our study was to test the efficacy of FMT in Clostridium difficile infection and Ulcerative Colitis refractory to standard medical treatments.

## Materials and method

We conducted a clinical prospective observational study of patients in the Clinical Emergency Hospital in Bucharest for 1 year. Consecutive patients with Clostridium difficile infection refractory to standard treatment or with more than one relapse and patients with ulcerative colitis non-responsive to anti-TNFa biological therapy (infliximab and adalimumab), were included in the study. Patient candidates for surgery and those who refused FMT were excluded from the study. 

The protocol of fecal administration was standardized. After signing the patient consent, the stool donators were chosen. They were selected from the close persons near the patients, with no recent infectious disease or cancers and who also had a normal diet. They were tested for HIV, HBV, HCV, and other bacterial or parasitic pathogens in the stool. The ones who tested positive for one of these diseases were excluded from the list of stool donors. 200-300 g of stools was mixed with 150-200 ml of saline until a homogenous solution was obtained. Most of the patients received this solution through colonoscopy (including terminal ileum) but those at risk of colonic perforation received the solution through nasojejunal tube (distal to the duodenojejunal angle of Treitz to limit reflux). The patient was first prepared with proton-pump inhibitors and oral vancomycin for 5 days. After the infusion of stool solution, the patient was hospitalized for as long as his general status allowed the discharge. The follow up duration was of 3 months.

Database variables were analyzed and included in Excel (Microsoft Office) according to the type of pathology, severity, number of stools per day before and after the FMT, the method of FMT infusion, the time to symptom resolution, adverse effects, number of tries before symptom resolution, relapse in the following 3 months. The statistical analysis was made in SPSS v.19.

## Results

A total of 33 patients were included in the study, of whom 28 had Clostridium difficile infection and 5 had ulcerative colitis. Of those with Clostridium difficile disease, 20 had a mild to moderate infection and the rest had a severe form. The mean number of stools per day was 5.6 for the whole group without any concordance with severity. Only 3 out of 28 had a recurrent infection. Twenty-seven patients received the infusion through colonoscopy and 1 through nasojejunal tube. The mean resolution time of symptoms was 2.3 days with a slight longer time for those having a severe form (2.7 days compared to 2 days) but without a statistical significance. One of the patients who had a severe form of infection needed another infusion. All the patients with Clostridium difficile had a resolution of symptoms with FMT, and, during the follow up period, only 1 had a relapse, which was successfully treated with oral Vancomycin. As for the adverse effects registered, 2 patients had fever (38 Celsius degrees) the first day after FMT. 

Of those with ulcerative colitis, 4 had a severe uncomplicated disease and 1 had a moderate form. The mean number of stools per day was 6.2 for this group. Three patients received the infusion through colonoscopy and 2 through nasojejunal route. Only one of the patients presented the resolution of symptoms after 1 week and only one infusion. Other 3 patients needed 3 to 4 infusions per patient until the symptoms disappeared and the mean time to symptoms resolution was 28 days. During the three-months follow up, one patient died, but there was no correlation with FMT. One of the remaining patients in the study relapsed and needed surgery. No adverse effects were registered in patients with ulcerative colitis.

## Conclusions

Fecal Microbiota transplantationationat for Clostridium difficile infection is a safe and efficient treatment with practically minimal costs and can be performed as an ambulatory procedure. Regarding the FMT for ulcerative colitis, the treatment showed promising results but future and extensive studies need to be done in order to prove its efficiency. 
